# Hydrogen Peroxide and Nitric Oxide Crosstalk Mediates Brassinosteroids Induced Cold Stress Tolerance in *Medicago truncatula*

**DOI:** 10.3390/ijms20010144

**Published:** 2019-01-02

**Authors:** Muhammad Arfan, Da-Wei Zhang, Li-Juan Zou, Shi-Shuai Luo, Wen-Rong Tan, Tong Zhu, Hong-Hui Lin

**Affiliations:** Ministry of Education Key Laboratory for Bio-Resource and Eco-Environment, College of Life Science, State Key Laboratory of Hydraulics and Mountain River Engineering, Sichuan University, Chengdu 610064, China; yuanmiao1892@163.com (D.-W.Z.); ljzou66@163.com (L.-J.Z.); loss0928@sina.com (S.-S.L.); wenrongtan1112@163.com (W.-R.T.); tzhu@ytu.edu.cn (T.Z.)

**Keywords:** brassinosteroids, cold stress tolerance, *Medicago truncatula*, hydrogen peroxide, nitric oxide, AOX capacity, PSII activity

## Abstract

Brassinosteroids (BRs) play pivotal roles in modulating plant growth, development, and stress responses. In this study, a *Medicago truncatula* plant pretreated with brassinolide (BL, the most active BR), enhanced cold stress tolerance by regulating the expression of several cold-related genes and antioxidant enzymes activities. Previous studies reported that hydrogen peroxide (H_2_O_2_) and nitric oxide (NO) are involved during environmental stress conditions. However, how these two signaling molecules interact with each other in BRs-induced abiotic stress tolerance remain largely unclear. BL-pretreatment induced, while brassinazole (BRZ, a specific inhibitor of BRs biosynthesis) reduced H_2_O_2_ and NO production. Further, application of dimethylthiourea (DMTU, a H_2_O_2_ and OH^−^ scavenger) blocked BRs-induced NO production, but BRs-induced H_2_O_2_ generation was not sensitive to 2-phenyl-4,4,5,5-tetramethylimidazoline-1-oxyl-3-oxide (PTIO, a scavenger of NO). Moreover, pretreatment with DMTU and PTIO decreased BL-induced mitochondrial alternative oxidase (AOX) and the photosystem capacity. However, pretreatment with PTIO was found to be more effective than DMTU in reducing BRs-induced increases in *V_alt_*, *V_t_*, and *MtAOX1* gene expression. Similarly, BRs-induced photosystem II efficiency was found in NO dependent manner than H_2_O_2_. Finally, we conclude that H_2_O_2_ was involved in NO generation, whereas NO was found to be crucial in BRs-induced AOX capacity, which further contributed to the protection of the photosystem under cold stress conditions in *Medicago truncatula*.

## 1. Introduction

Plants are exposed to unfavorable environmental conditions during their life cycle, which leads to a reduction in productivity. To survive in adverse conditions, plants develop several complex mechanisms [1,2]. Low-temperature is one of major abiotic stress factors affecting plant growth and production. Plant species growing in both temperate and cold zones have developed an effective mechanism to enhance their freezing tolerance [3]. Several physiological, biochemical and molecular changes occur during cold stress. These changes include accumulation of proline, glycine betaine, sugar [4], and scavenging of reactive oxygen species (ROS) [5].

Brassinosteroids (BRs) are a class of plant steroid hormone that play a diverse role in floral organ elongation, seed germination, and vascular development [6]. In addition, BRs enhance tolerance to various stresses such as temperature variability, drought, salinity, heavy metals, and pathogenic infections [7,8]. Previous studies revealed that BRs could enhance stress tolerance by regulating the expression of several genes [7,9]. For instance, BRs-induced cold stress tolerance in cucumber, mainly through regulating defense related genes [10]. Further, BRs-induced stress tolerance has been associated with alteration of malondialdehyde MDA contents and antioxidant metabolism [11].

Plant responses to environmental stresses are often linked with the accumulation of reactive oxygen species (ROS) [12]. Among several ROS, hydrogen peroxide (H_2_O_2_) is considered to be the most stable molecule, which is involved in many defense related mechanisms [13]. Hydrogen peroxide (H_2_O_2_) generation occurs in the cell membrane because of nicotinamide adenine dinucleotide phosphate (NADPH) oxidase. Previously, it has been reported that respiratory burst oxidase homologues (*RBOH*) genes are crucial in H_2_O_2_ production [14,15]. It is well established that H_2_O_2_ acts as a stress signal in plants [16,17]. However, non-toxic levels must be maintained in a delicate balancing between H_2_O_2_ production and scavenging. Furthermore, several previous reports also confirmed nitric oxide’s (NO) role as an endogenous signaling molecule during abiotic stress conditions [18,19]. Nitrate reductase (NR) and nitric oxide synthase (NOS) have been reported as two enzymatic sources [20,21]. Moreover, non-enzymatic pathways may participate in NO production [18,20]. BRs role in the accumulation of NO has already been discussed [22]. Previously, several studies have demonstrated that alternative oxidase (AOX) plays an important role during environmental stress tolerance [23,24]. Also, there is much evidence available on AOX’s role in scavenging mitochondrial ROS [25,26]. In addition, AOX involvement in the enhancement of photosystem damage has also been reported [27,28].

Legumes are considered as an important source for grain and forage. These are grown on around 15% of the earth’s arable surface [29]. We selected *Medicago truncatula* because it serves as a model plant for molecular studies, especially for legumes. The availability of a complete genome and short life cycle distinguished it for this research purpose [30,31]. Several recent studies confirmed BRs role in abiotic stress tolerance [7,9]. To date, few reports in the literature have focused on the H_2_O_2_ and NO involvement as signaling molecules in BRs-mediated plant stress tolerance [32,33,34]. Therefore, we hypothesized that there might be a link between H_2_O_2_ and NO in BRs-induced abiotic stress tolerance. Here, this hypothesis was tested and our experiments results demonstrated that H_2_O_2_ and NO crosstalk involved in BRs-induced plant tolerance to environmental stress.

## 2. Results

### 2.1. Brassinosteroids (BRs) Treatment Results in the Improvement of Cold Stress Tolerance

**Figure 1:**
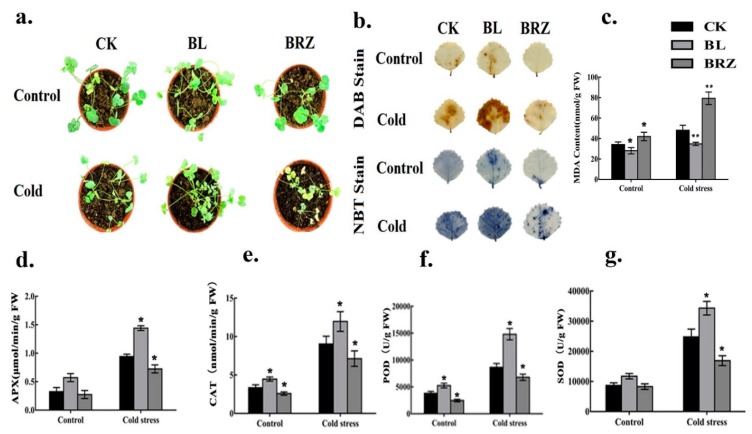
Effects of BRs treatment on cold stress tolerance in *M. truncatula* plants. Plants were pretreated with 1 μM BL or 1 μM BRZ or water (CK) to test BRs role. Both control (non-stressed) and cold stress (4 °C) plants’ leaf tissues were collected after 72 h for the detection of enzymes activities and MDA contents. The cold-treated plants photographed after 3 days (**a**), detection of ROS sites in leaves treated with BL (**b**), determination of MDA contents (**c**), and antioxidant enzymes activities (**d**–**g**). Bars represent mean and standard deviation of values obtained from three biological replicates. Significant differences (*p* < 0.05) are denoted by asterisks.

To investigate the role of brassinosteroids (BRs) in cold stress tolerance, some important physiological parameters were monitored after 72 h. Under control conditions (non-stressed), plants pretreated with brassinolide (BL) exhibited less difference in phenotypes compared to brassinazole (BRZ) or water (CK), where leaves of the BL-pretreated plants appeared better (Figure 1a). However, it was easy to distinguish the different phenotypes of all treatments under cold stress. Here, BRZ-pretreated plant leaves showed more chlorosis symptoms (yellowing of leaves) compared with BL or CK (Figure 1a). Overall, BL-pretreated plants exhibited improved phenotypes in both control and cold-acclimation (Figure 1a). Further, the reactive oxygen species (ROS) were observed in BRs-induced cold stress tolerance. Hydrogen peroxide (H_2_O_2_) and the superoxide (O_2_^−^) anion did not accumulate much in leaves under non-stressed conditions (control). The staining sites increased in leaves after cold treatment, while more ROS were accumulated in BL-pretreated leaves than BRZ-pretreated or CK (Figure 1b). To assess the degree of damage in plants, MDA contents were investigated. BL-pretreated plants showed lower levels of MDA contents compared with BRZ-pretreated or CK plants (Figure 1c). Further, we analyzed antioxidants that protect the cell from oxidative damage by scavenging ROS. Compared to the non-stressed (control), the activities of APX, CAT, POD, and SOD were increased after treatment with cold (Figure 1d–g). The activities increased in BL-pretreated plants more than water (CK) or BRZ-pretreated plants.

### 2.2. BRs Regulate Cold-Related Genes’ Expression

**Figure 2:**
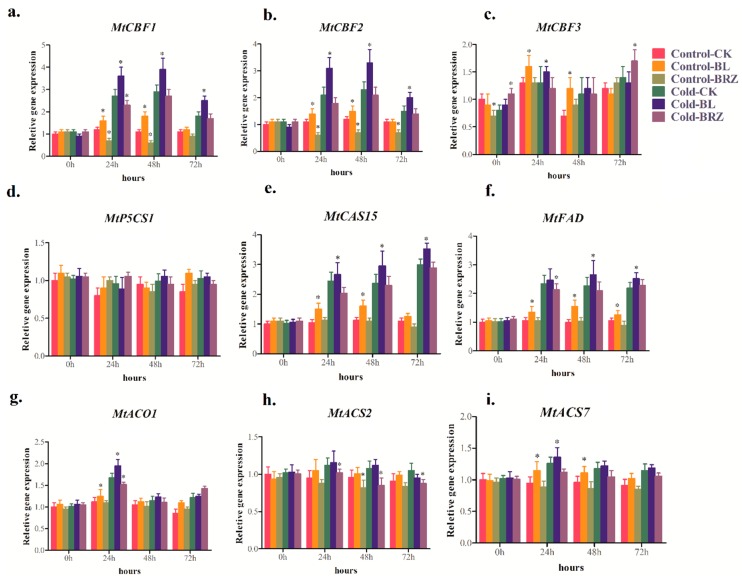
Response of several cold-related genes (**a**–**i**) was determined using quantitative RT-PCR. Plants were pretreated with 1 μM BL or 1 μM BRZ or water (CK) to investigate BRs effect. Bars represent the mean and standard deviation of values obtained from three biological replicates. Significant differences (*p* < 0.05) are denoted by asterisks.

To analyze the underlying mechanisms for BRs-induced cold stress tolerance, we examined the effects of BRs levels on the expression of several genes involved in the cold-defense response. Cold-stress responsive genes targeted by dehydration-responsive element protein (DREB1)/C-repeat (CRT)-binding factor (CBF) was involved in the cold stress response. Furthermore, the expression of *CAS* genes was positively correlated with cold stress tolerance. Expression of 1-aminocyclopropane-1-carboxylate (ACC) synthase (*ACS*) and ACC oxidase (*ACO*) genes were involved in ethylene production [35]. Fatty acid desaturase (FAD) was involved in desaturation of the fatty acids. *FAD* genes’ expression helped to understand the benefits of fatty acid desaturation in plant stress responses [36]. The expression of *MtCBFs*, *MtP5CS1*, *MtCAS15*, *MtFAD*, *MtACO1*, *MtACS2*, and *MtACS7* genes were monitored. A rapid increase in transcripts of *MtCBF1* (Figure 2a), *MtCBF2* (Figure 2b), *MtCBF3* (Figure 2c), *MtCAS15* (Figure 2e), *MtFAD* (Figure 2f), *MtACO1* (Figure 2g), *MtACS2* (Figure 2h), and *MtACS7* (Figure 2i) was observed in BL-pretreated plants after 24 and 48 h of cold acclimation (4 °C), and almost had little change in BRZ-pretreated plants compared with water-pretreated plants (CK). Under cold-acclimated conditions (4 °C), compared with water-pretreated plants (CK), expression of these genes was up-regulated upon treatment with BL, whereas they were down-regulated after BRZ treatment. Furthermore, *P5CS1* was found to be involved in the control of proline levels during and after osmotic stress [37]. However, expression of *MtP5CS1* in BL-pretreated plants was sustained under both control (non-stressed) and cold-stress conditions (Figure 2d).

### 2.3. BRs-Treatment Induced Hydrogen Peroxide and Nitric Oxide Accumulation in Leaves

**Figure 3:**
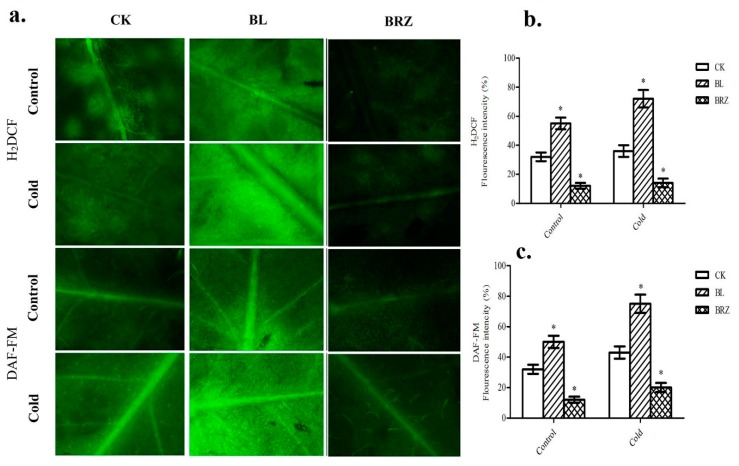
BRs’ role in the generation of hydrogen peroxide (H_2_O_2_) and nitric oxide (NO). *M. truncatula* plants pretreated with 1 μM BL or 1 μM BRZ or water (CK). Images were taken after 24 h of chemical treatment using a fluorescence microscope (Scale bars = 75 μm) (**a**). Quantitative measurements of H_2_O_2_ level (**b**) and NO level (**c**) in young leaves. Bars represent mean and standard deviation of values obtained from three biological replicates. Significant differences (*p* < 0.05) are denoted by asterisks.

To investigate whether BRs-induce hydrogen peroxide and nitric oxide accumulation, we detected the fluorometric assay using probe H_2_DCF-DA and DAF-FM DA, respectively, with fluorescence microscope. As shown in Figure 3, pretreatment with BL accelerated both H_2_O_2_ and NO accumulation, which suggested that the significant increase of H_2_O_2_ and NO level was attributed to the BL treatment. More interestingly, after cold stress (4 °C) treatment, the fluorescence level increased compared to control (non-stressed) conditions. However, BL-induced H_2_O_2_ and NO accumulation was significantly higher compare to water (CK) and BRZ. Noticeably, elevated H_2_O_2_ and NO levels were observed in both control (non-stressed) and cold treatment leaves (Figure 3), suggesting that H_2_O_2_ and NO may be involved in BR-mediated cold stress tolerance.

### 2.4. Relationship between BRs-Induced Hydrogen Peroxide and Nitric Oxide Accumulation

**Figure 4:**
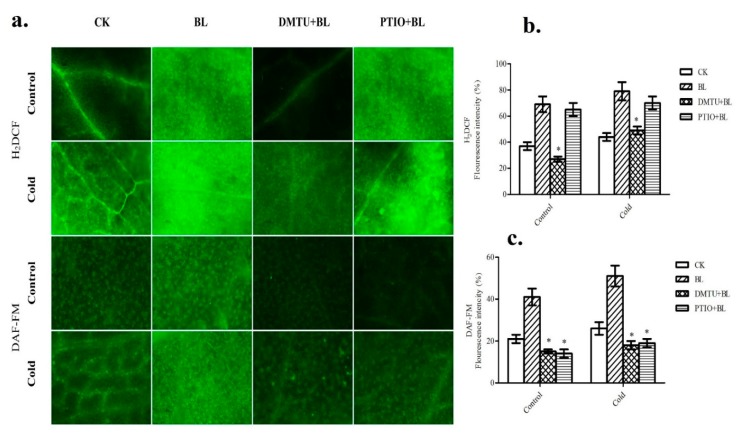
The relationship between BRs-induced H_2_O_2_ and NO accumulation under cold stress conditions. Images were taken after 24 h of chemicals treatment using a fluorescence microscope (Scale bars = 75 μm) (**a**). Quantitative measurements of H_2_O_2_ level (**b**) and NO level (**c**) in young leaves. Bars represent mean and standard deviation of values obtained from three biological replicates. Significant differences (*p* < 0.05) are denoted by asterisks.

We then investigated the potential link between H_2_O_2_ and NO in BRs-induced cold stress tolerance. Here, DMTU (a H_2_O_2_ and OH^−^ scavenger) and PTIO (a scavenger of NO) were used to understand the relationship between H_2_O_2_ and NO, respectively. As shown in Figure 4, pretreatment with BL enhanced the H_2_O_2_ and NO accumulation that could be observed by the fluorescence, while DMTU + BL and PTIO + BL scavenged the BL-induced H_2_O_2_ and NO production. Notably, pretreatment with DMTU + BL significantly declined the BL-increased NO fluorescence levels in both the control (non-stressed) and cold stress (4 °C) leaves. However, pretreatment with PTIO + BL had little effect on BL-induced H_2_O_2_ production, both in control (non-stressed) and cold stress plant leaves (Figure 4). 

### 2.5. The Role of Hydrogen Peroxide and Nitric Oxide in BRs-Induced Alternative Respiratory Pathway

**Figure 5:**
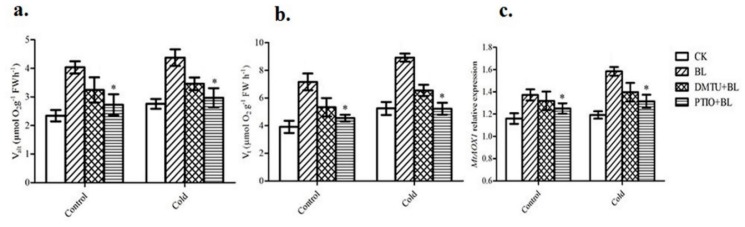
Effect of H_2_O_2_ and NO in BRs-induced alternative respiratory pathway. Young leaves from the same position of each plant were harvested and used for the determination of alternative respiration (*V_alt_*) (**a**), total respiration (*V_t_*) (**b**), and *MtAOX1* expression (**c**). Bars represent mean and standard deviation of values obtained from three biological replicates. Significant differences (*p* < 0.05) are denoted by asterisks.

To investigate the effects of BR-induced H_2_O_2_ and NO production on the BR-induced alternative respiratory pathway, *M. truncatula* leaves were pretreated with DMTU (a H_2_O_2_ and OH^−^ scavenger) or PTIO (a scavenger of NO), and then exposed to BL treatment. Pretreatment with PTIO substantially reduced the BRs-induced increases in *V_alt_*, *V_t_*, and *MtAOX1* expression, whereas pretreatment with DMTU had less of an effect on *V_alt_*, *V_t_*, and *MtAOX1* expression when compared with BL-pretreated plants (Figure 5a–c). 

### 2.6. Hydrogen Peroxide and Nitric Oxide Involved in BRs-Induced Defense of Photosystem

**Figure 6:**
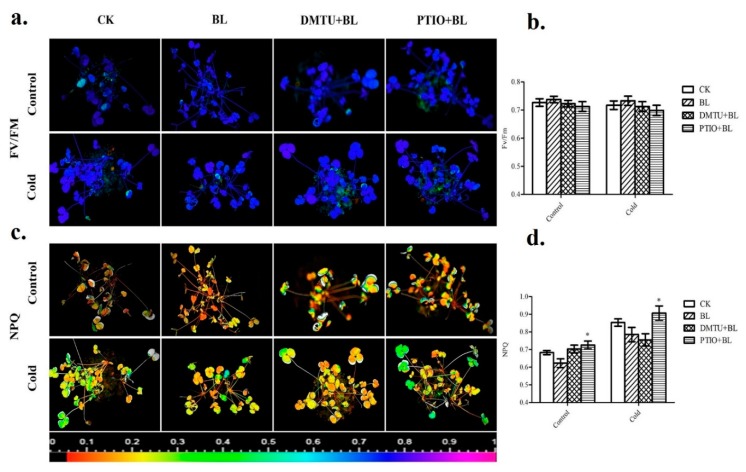
Chlorophyll fluorescence parameters were determined with an imaging PAM (IMAG-MAXI; HeinzWalz). Images and values of the *F*_V_/*F*_M_ (**a**,**b**) and NPQ (**c**,**d**) in various treatments. The false color code depicted at the bottom of the image ranged from 0 (black) to 1.0 (purple). All the leaf samples were collected at the same time every day. Bars represent mean and standard deviation of values obtained from three biological replicates. Significant differences (*p* < 0.05) are denoted by asterisks.

To determine whether H_2_O_2_ and NO, which were induced by BRs, play a critical role in BRs-induced cold stress tolerance, we further investigated their relationship. We analyzed the effects of DMTU (a H_2_O_2_ and OH^−^ scavenger) and PTIO (a scavenger of NO) on BL-induced tolerance to cold stress. These results suggested that in the BL-pretreated plants, *F*_V_/*F*_M_ was higher than those in water-pretreated plants (CK) under a cold stress condition (4 °C); in other words, BL pretreatment alleviated the cold-induced decline of *F*_V_/*F*_M_. (Figure 6a,b). However, pretreatment with DMTU or PTIO blocked the BL-induced stress tolerance. These results showed that PTIO pretreated plants had lower *F*_V_/*F*_M_ than DMTU pretreated plants under cold stress conditions (Figure 6a,b). Moreover, NPQ was significantly higher in PTIO pretreated plants than those in DMTU pretreated plants under stress conditions (Figure 6c,d). 

## 3. Discussion

### 3.1. Involvement of BRs in Cold Stress Tolerance

Recent studies reported that BRs are involved in plant tolerance to environmental stress [7,8]. However, mechanisms of BR-enhanced abiotic stress tolerance remain to be determined in detail. Here, we found that BRs effectively induced physiological, biochemical, and molecular mechanisms (Figure 1 and Figure 2). As shown in Figure 1a, the *M. truncatula* plant pretreated with BL demonstrated better phenotypes than water or BRZ, where more leaves turned yellow under cold stress condition (Figure 1a). ROS production during stressful environment considered as a common cellular phenomenon. Previous studies reported that ROS may act as a defense signal during a stressful environment [25,38]. Here, we found that BRs treatment enhanced ROS production of superoxide (O_2_^−^) anion and hydrogen peroxide (H_2_O_2_), which may also involve in transferring a defense signal (Figure 1b). Moreover, BRs were found to be involved in alleviating oxidative damage. The MDA contents significantly decreased in BL-pretreated plants compared to BRZ- pretreated plants under cold stress (Figure 1c). The decline in the levels of MDA content suggests BRs mitigated the damaging effect of environmental stresses. Recent studies also showed that BRs are involved in alleviating oxidative damage [25,39]. Previous studies indicated that BRs enhanced the antioxidative enzymes activities [39,40,41]. In our study, we found that BRs elevated antioxidative enzymes activities (Figure 1d–g), which may involve cold stress tolerance. In the present study, we found that BL-pretreatment significantly enhanced ROS generation (Figure 1b) and antioxidative enzymes activities (Figure 1d–g), while decreasing MDA content (Figure 1c), suggesting BRs enhanced cold stress tolerance. 

Several molecular processes are altered when plants suffer from environmental stress. Here, BR-mediated enhancement of cold-related genes expression (Figure 2) may be involved in cold tolerance. Among them, changes in expression pattern of *CBFs* and *COR* genes are frequently involved in cold stress [7,42]. Here, BL-pretreated plants enhanced the expression of *CBFs* and *COR* genes and had little change in the BRZ-pretreated plants compared with water-pretreated plants (CK). The involvement of ethylene in response to low temperature has been reported [35,42]. Hansen et al., concluded that BRs positively influence ethylene biosynthesis through the regulation of ACC synthase (*ACS*) and ACC oxidase (*ACO*) genes activity [43]. In this study, we found that ethylene regulated genes *MtACO1*, *MtACS2*, and *MtACS7* were also induced in plants pretreated with BL than BRZ or water (Figure 2g–i). These results suggest that BRs may enhance cold stress tolerance by up-regulating expression of *MtACO1*, *MtACS2*, and *MtACS7* genes. These observation indicated that BRs might participate in a variety of physiological functions that activate the defense system and enable plants to acquire resistance.

### 3.2. BRs-Induced H_2_O_2_ and NO Generation in Medicago truncatula

Both H_2_O_2_ and NO have been shown to play a key role in plants during environmental stresses [18]. Numerous studies have reported that H_2_O_2_ and NO production in stressful environmental conditions occur in a similar or parallel relation [44,45]. Recent studies revealed BRs role in H_2_O_2_ and NO production [22,46,47]. Although H_2_O_2_ and NO was found to be involved in BRs-induced stress tolerance, the relationship between these two molecules in BRs-mediated cold stress signaling remain unclear. Therefore, it was of interest to explore the relationship between these two molecules in BR-mediated cold stress tolerance. As shown in Figure 4, pretreatment with DMTU blocked BRs-induced increase in H_2_O_2_ levels and substantially reduced BRs-induced increase in NO levels of *M. truncatula* leaves. These results suggested that H_2_O_2_ involved in BRs-induced NO generation. On the other hand, PTIO pretreated leaves could not significantly affect the increase of H_2_O_2_ after BRs treatment (Figure 4). The present study revealed that H_2_O_2_ involved in BRs-induced NO generation under cold stress.

### 3.3. Involvement of H_2_O_2_ and NO in BRs-Induced Alternative Pathway and PSII

The alternative oxidase (AOX) plays a crucial role in stress resistance. Previous report revealed that AOX is involved in development of freezing tolerance [16,48]. Several plant hormones, such as salicylic acid [49], Jasmonic acid [50] and ethylene [51], is found to be involved in AOX induction. In addition, another study reported that *AOX1* expression was enhanced by application of abscisic acid (ABA), while declined by the *abi4* mutant [52]. In our study, we showed that BRs could also induce AOX, especially under cold stress condition in *M. truncatula*. Previously, It has been revealed that H_2_O_2_ and NO are involved in BRs-induced AOX capacity [32,34]. However, the relationship between H_2_O_2_ and NO has never been explored. In this study, we have provided evidence that pretreatment with PTIO is more effective than DMTU for the reduction of BRs-induced increase in *V_alt_*, *V_t_*, and *MtAOX1* gene expression (Figure 5a–c). These results suggested that BRs-induced alternative respiratory pathway, which was more in a NO-dependent manner than H_2_O_2_ and may played a critical role in BL-induced cold stress tolerance.

Further, our results suggested that the enhanced AOX activity by BRs contributed to the protection of the photosystem under stress conditions (Figure 6a–d), similar to previous reports [52]. Previous findings indicated that the mitochondrial AOX pathway may be involved in the protection of plants from photosystem damage by scavenging ROS [53]. Similarly, reference [27] reported that AOX was particularly involved in alleviating photosystem damage under stress conditions. Our results indicated that BRs-induced PSII efficiency under environmental stress condition may depend on NO production more necessarily than H_2_O_2_ (Figure 6a–d). Thus, our results suggest that NO is essential in BRs-induced AOX capacity, which further contribute to the protection of the photosystem under cold stress conditions in *Medicago truncatula*.

**Figure 7:**
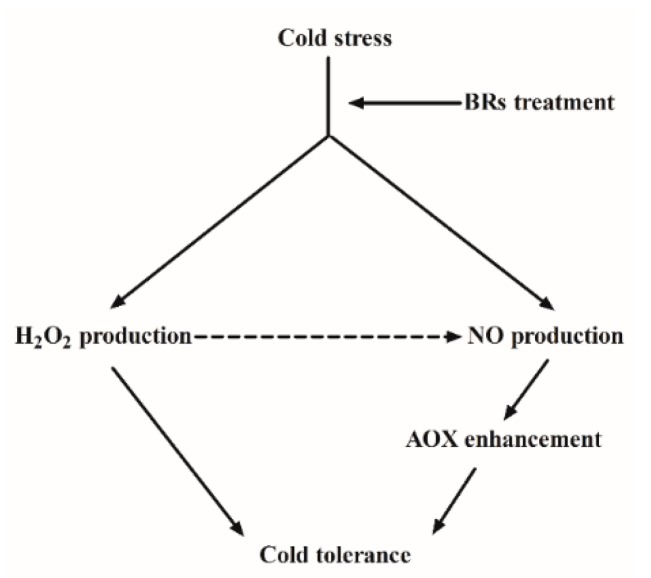
Model illustrating the involvement of H_2_O_2_ and NO in BRs-induced cold stress tolerance.

In conclusion, we have revealed that H_2_O_2_ and NO are involved in the BRs-mediated cold defense signaling pathway. The possible signaling pathway for BRs-mediated cold stress tolerance is summarized in Figure 7. BRs enhanced the H_2_O_2_ and NO production level in *M. truncatula* under cold stress. Furthermore, H_2_O_2_ produced NO, which plays a crucial role in the enhancement of AOX capacity. Improved AOX capacity further contributed to alleviating the photosystem damage under cold stress conditions. Furthermore, BRs-mediated signaling pathway in response to cold stress improves our understanding about plant response mechanisms to other environmental stresses.

## 4. Materials and Methods

### 4.1. Plant Material, Growth Condition, and Chemical Treatment

The *Medicago truncatula* “108-R” plant was used in this cold stress experiment. First, seeds were scarified with concentrated H_2_SO_4_ for 5 min and then washed with distilled water. Later, these seeds were shifted in petri-plates on moistened filter paper at 4 °C for three nights. After germination, seedlings were transplanted into pots containing perlite and sand (3:1). The plants were grown in a greenhouse under controlled conditions at 25 °C with a 16-h-light/8-h-dark cycle (100 μmol·m^−2^·S^−1^). The seedlings used in the experiments were 4 weeks old. Brassinolide (BL, the most active BRs) and brassinazole (BRZ, a specific inhibitor of BRs biosynthesis) were purchased from Wako Pure Chemical Industries (http://www.wakochem.co.jp/english) and Santa Cruz Biotechnology (http://www.scbt.com), respectively. Dimethylthiourea (DMTU, a H_2_O_2_ and OH^−^ scavenger) and 2-phenyl-4,4,5,5-tetramethylimidazoline-1-oxyl-3-oxide (PTIO, a scavenger of NO) were purchased from Sigma-Aldrich (http://www.sigmaaldrich.com). The chemical solutions were prepared in water containing 0.02% *v*/*v* Tween 20. Following three different groups of treatments were used to manipulate BRs level in *M. truncatula*: Brassinolide (BL), brassinazole (BRZ), and CK (water). For chemical treatment, plants were pretreated using foliar spraying with 1 μM BL or 1 μM BRZ. Distilled water containing 0.02% (*v*/*v*) Tween 20 was used as a CK treatment. To investigate the role of H_2_O_2_ and NO in BL-induced cold stress tolerance DMTU + BL and PTIO + BL were used, respectively. First plants were pretreated with 5 mM DMTU or 200 μM PTIO and 8 h after pretreatment, then the plants were sprayed with 1 μM BL for another 16 h. The plants were then exposed to cold stress (4 °C) at 24 h after the chemical treatment. This method was adopted from previous research [32], with some modifications. 

### 4.2. Quantification of Superoxide (O_2_^−^) Anion, Hydrogen Peroxide (H_2_O_2_), and Malondialdehyde (MDA)

ROS, such as superoxide and hydrogen peroxide, accumulation sites were visualized in 4-week-old plants using traditional staining techniques. The accumulation of superoxide anion and hydrogen peroxide determined using nitroblue tetrazolium (NBT) and 3,3′-diaminobenzidine (DAB) stains, respectively [24]. *M. truncatula* leaves were first excised at the base with a razor blade and then vacuum infiltrated in solutions with NBT (0.5 mg·mL^−1^) for 3 h or DAB (2 mg·mL^−1^) for 12 h. Leaves were then decolorized in boiling ethanol (95%) for 30 min. At least five single leaves were used for each staining treatment.

The first fresh trifoliate leaves were collected to measure lipid peroxidation. The contents of MDA were measured following the manufacturer instructions (Comin Biotechnology Co., Ltd. Suzhou, China). The level of lipid peroxidation was checked using a malondialdehyde (MDA) content assay kit (Comin Biotechnology Co., Ltd. Suzhou, China), following the manufacturer instructions. The principle was based on the thiobarbituric acid (TBA) method [54]. The absorbance of the supernatant was recorded at 532 and 600 nm. The MDA content was calculated using the following formula: MDA content (nmol/g FW) = 25.8 × ΔA ÷ W (W: fresh weight of sample; ΔA: A_532_ − A_600_).

### 4.3. Determination of Antioxidant Enzymes Activities

The first fresh trifoliate leaves were detached to measure enzymes activities. The activities of APX, CAT, POD, and SOD were measured following the manufacturer instructions (Comin Biotechnology Co., Ltd. Suzhou, China).

Ascorbate peroxidase (APX) activity was determined based on monitoring the decrease in absorbance at 290 nm as ASA was oxidized [55]. The absorbance change of reaction mixture was measured at 290 nm for 10 s and 130 s, denoted by A1 and A2, respectively. One unit was defined as the oxidation of 1 μmol ASA in the reaction system per gram fresh weight per minute. CAT activity was calculated using the following formula: APX activity (μmol/min/g FW) = 1.79 × ΔA ÷ W (W: fresh weight of sample, g; ΔA = A_1_ − A_2_). 

The catalase (CAT) activity was determined based on monitoring the absorbance of H_2_O_2_ at 240 nm [56]. The absorbance change of the reaction mixture was measured at 240 nm for 10 s and 70 s, denoted by A1 and A2, respectively. One unit was defined as the degradation of 1 nmol H_2_O_2_ in the reaction system per gram fresh weight per minute. The CAT activity was calculated using the following formula: CAT activity (nmol/min/g FW) = 678 × ΔA ÷ W (W: fresh weight of sample, g; ΔA = A_1_ − A_2_).

The peroxidase (POD) activity was determined based on the guaiacol method [57], following the manufacturer’s protocol. The reaction mixture absorbance change was measured at 470 nm for 30 s and 90 s, which were denoted as A1 and A2, respectively. One POD activity unit was defined as the increase of 0.01 of record absorbance in a 1 mL reaction system per gram fresh weight per minute. The POD activity was calculated according to the following formula: POD activity (U/g FW) = 7133 × ΔA ÷ W (W: fresh weight of sample, g; ΔA = A_2_ − A_1_). 

The superoxide dismutase (SOD) activity was determined using xanthine and xanthine oxidase (XO) to generate O_2_^−^, which reacts with (2-(4-iodophenyl-3-(4-nitrophenyl)-5-phenyltetrazolium; INT) to form a red azan dye. The SOD activities were recognized by calculating the percentage of inhibition [57]. The absorbance was measured at 560 nm using a spectrophotometer (Thermo Scientific Multiskan GO). The percentage of inhibition was calculated = [A (control) − A (experiment)] ÷ [A (control)] × 100%. The percentage of inhibition of 50% in the reaction system was defined as one unit. Finally, the SOD activity was calculated using the following formula: SOD activity (U/g FW) = 11.4 × percentage of inhibition ÷ (1 − percentage of inhibition) ÷ W × dr (W: fresh weight of sample, g; dr: dilution ratio).

### 4.4. Measurement of Endogenous H_2_O_2_ and NO

For fluorescence microscopy, NO and H_2_O_2_ were visualized using Diaminofluorescein-FM diacetate (DAF-FM-DA) and 2′,7′-dichlorofluorescein diacetate (H_2_DCF-DA) probes, respectively (Sigma-Aldrich). Briefly, leaf dices were first loaded with 15 μM DAF-FM-DA for 30 min or 50 μM H_2_DCF-DA for 10 min in Tris/KCl loading buffer (pH 7.2). The process was performed in darkness at 25 °C. Further, leaves were washed three times (5 min each) using Tris/KCl loading buffer (10 mM Tris and 50 mM KCl, pH 7.2), and visualized under a TE2000-U fluorescence microscope (490 nm excitation; 515 nm emission) (Nikon, Tokyo, Japan). Treatments were repeated at least five times. NO and H_2_O_2_ contents were also detected using a Griess reagent (Sigma-Aldrich) and an Amplex red hydrogen peroxide/peroxidase assay kit (Invitrogen), respectively [33].

### 4.5. Respiration Measurements

Respiratory oxygen consumption was measured using Clark-type electrodes (Hansatech, King’s Lynn, U.K.), according to Xu et al., (2012) after 24 h of cold stress. Around 0.05 g of young single leaves were first cut into small pieces, and then pretreated with 5 mL deionized water for 15 min in order to eliminate wound induced respiration. These measurements were performed at 25 °C in 2 mL (final volume) of phosphate buffer (pH 6.8), and the cuvette was tightly closed to prevent diffusion of oxygen from the air. Inhibitors of the cytochrome pathway (1 mM KCN) and the alternative pathway (0.5 mM n-propyl gallate, nPG) were used. The total respiration (*V_t_*) was defined as the O_2_ uptake rate by *M. truncatula* leaves without any inhibitor. Then, 1 mM KCN was added to obtain the O_2_ uptake rate, defined as *V*_0_. The residual respiration (*V_res_*) was estimated by measuring the rate of oxygen uptake in the presence of both 1 mM KCN and 0.5 mM nPG. The individual capacity of the cytochrome pathway (*V_cyt_)* and the alternative pathway (*V_alt_)* was calculated using the following formulas: *V_cyt_ = Vt* − *V*_0_; *V_alt_ = V*_0_ − *V_res_*. The *V_res_* in our experiment was always low and was negligible relative to other respirations. Therefore, the *V_res_* was not shown [34].

### 4.6. Chlorophyll Fluorescence Analysis

In this experiment, an imaging pulse amplitude modulated (PAM) fluorometer (IMAG-MINI; Heinz Walz, Effeltrich, Germany) was used to determine chlorophyll fluorescence. For the measurement of the maximal quantum efficiency of photosystem II (*F*_V_/*F*_M_), plants were first dark-adapted for 30 min. Minimal fluorescence (*F*_0_) was measured during the weak measuring pulses and maximal fluorescence (*F*_M_) was measured using a 0.8 s pulse light at about 4000 μmol·m^−2^·s^−1^. An actinic light source was then applied to obtain steady-state fluorescence yield (*F_S_*), after which a second saturation pulse was applied for 0.7 s to obtain a light-adapted maximum fluorescence (*F*_M_′). The *F*_V_/*F*_M_ and NPQ/4 were calculated as (*F*_M_ − *F*_0_)/*F*_M_ and (*F*_M_ − *F*_M_′)/*F*_M_′, respectively [58,59].

### 4.7. RNA Extraction and Quantitative Real-Time PCR (qRT-PCR) Analysis

In this experiment, total RNA was extracted from the first fresh trifoliate leaves according to previously described method [60], with some modifications. The cDNA was amplified using a SYBR Premix Ex Taq (TaKaRa Bio Inc., Dalian, China). All genes expression level was measured by quantitative real time PCR (qRT-PCR). The threshold cycle (*C*_t_) was defined as the PCR cycle at which a statistically significant increase in reporter fluorescence was first detected, and it was used as a measure for the starting copy numbers of the target gene. *MtActin* gene was used as an internal control. At least three biological replicates were performed for each sample, and three technical replicates were analyzed for each biological replicate. The primers used are listed in Table S1.

### 4.8. Statistical Analysis

Data from experiments with three or more mean values were statistically analyzed using one way ANOVA. The difference was considered to be statistically significant at *p* < 0.05.

## Supplementary Materials

Supplementary materials can be found at http://www.mdpi.com/1422-0067/20/1/144/s1.

## Abbreviations

BRs	Brassinosteroids
BL	Brassinolide
BRZ	Brassinazole
DMTU	Dimethylthiourea
PTIO	2-phenyl-4,4,5,5-tetramethylimidazoline-1-oxyl-3-oxide
