# Supplementary material for: Hydrogen Peroxide and Nitric Oxide Crosstalk Mediates Brassinosteroids Induced Cold Stress Tolerance in Medicago truncatula
Source: Int J Mol Sci. 2019 Jan 2;20(1):144. doi: 10.3390/ijms20010144 (PMC6337477; doi:10.3390/ijms20010144)
Supplement: Supplementary file 1 [file ijms-20-00144-s001.pdf]

Table S1. Primer sequences used for real-time quantitative RT-PCR

Gene	Accession	F-primer	R-primer
MtAOX1	Medtr5g026620	GAATGCTGCTTCACTG	CTCGTCCTTGGTAATGT
MtCBF1	EU139866	GATTTGGCTTGGAACATA	ACAGCATCCCTATCACTC
MtCBF2	EU139867	GAAACTTAGATAAATGGGTG	GTAGCAGAGGTGGAAATAG
MtCBF3	EU139868	ACAATGACAATGACAATGACAAT	AAAAACTCCATAGTGATACTTCCTC
MtP5CS1	AJ278818	TGATTTGTGAGTTTTGCTAC	CTCATAAGCAATTACAGAGC
MtCAS15	EU139869	GGTGATGCCCTTCACATA	TCCCCATGGTGTCCTTCC
MtFAD	A0A072V8W8_MEDTR	CATTTACTCAGATCGTGAAC	CCACATGAGTGTCTGTAATA
MtACO1	XM003601545	CCAAAGGGCTAGAGGCTGTTC	GGTAGGTGACGCAAATGGAAA
MtACS2	AY0620022	TGCCTACACCTTACTATCCAG	TCTGTCCATAACTGTGCCTAA
MtACS7	XM003620831	CGGTCTACCAGGTTTCAGAGT	TCAAGCATTCAATTCCAGCAC
MtACTIN	XM003621972	TGACCTTACCGATTTCTTG	CTGCTGCTTCCATTCCTAT
